# Spectral collapse in multiqubit two-photon Rabi model

**DOI:** 10.1038/s41598-021-84934-y

**Published:** 2021-03-08

**Authors:** C. F. Lo

**Affiliations:** Institute of Theoretical Physics and Department of Physics, The Chinese University of Hong Kong, Shatin, New Territories Hong Kong

**Keywords:** Optical physics, Quantum physics

## Abstract

We have shown that the smallest possible singel-qubit critical coupling strength of the N-qubit two-photon Rabi model is only 1/N times that of the two-photon Rabi model. The spectral collapse can thus occur at a more attainable value of the critical coupling. For both of the two-qubit and three-qubit cases, we have also rigorously demonstrated that at the critical coupling the system not only has a set of discrete eigenenergies but also a continuous energy spectrum. The discrete eigenenergy spectrum can be derived via a simple one-to-one mapping to the bound state problem of a particle of variable effective mass in the presence of a finite potential well and a nonlocal potential. The energy difference of each qubit, which specifies both the depth of the finite potential well and the strength of the nonlocal potential, determines the number of bound states available, implying that the extent of the incomplete spectral collapse can be monitored in a straightforward manner.

## Introduction

The two-photon Rabi model is the simplest model describing the nonlinear two-photon process in light-matter interacting systems. Due to the weak coupling of the two-photon process in different physical setups, its applications have been rather limited. Recent advancement in quantum technology has transformed the situation dramatically and made the applications of the two-photon Rabi model feasible even in the strong coupling regime^1–11^. For instance, Felicetti et al.^1,4^ pointed out that a trapped-ion scheme allows one to experimentally investigate two-photon interactions in unexplored regimes of light-matter coupling, and that a circuit quantum electrodynamics scheme enables us to implement a nondipolar ultrastraong two-photon interaction between a flux qubit and a bosonic mode supported by SQUID. In spite of its simplicity, the two-photon Rabi model displays a counter-intuitive feature, namely the “spectral collapse”, when the coupling strength $\epsilon$ of the light–matter interaction goes beyond a critical value $\epsilon _{c}$^12–23^. That is, while the model has a discrete eigenenergy spectrum for $\epsilon <\epsilon _{c}$, no normalizable eigenstate exists in the Hilbert space spanned by the photon number states for $\epsilon >\epsilon _{c}$. At the critical coupling $\epsilon _{c}$ the spectral collapse is, however, found to be incomplete for the eigenenergy spectrum of the model consists of both a set of discrete energy levels and a continuous energy spectrum^24^. The number of bound states available is determined by the energy difference $\omega _{0}$ between the two atomic levels so that the extent of the incomplete spectral collapse can be monitored straightforwardly.

In a recent paper, via investigating a generalization of the two-photon Rabi model, in which the two-photon coupling is replaced by a full quadratic coupling, Lo^25^ has demonstrated that the critical coupling strength is reduced by half from that of the two-photon Rabi model. Similar to the two-photon Rabi model, at the critical coupling the discrete eigenenergy levels of the generalized two-photon Rabi model can be derived from a simple quantum mechanical bound state problem, namely a particle of variable effective mass moving in a finite potential well specified by the square of the “Lorentzian function”^25^. In addition, Felicetti and his coworkers^1,4,26^ have pointed out that the two-photon Dicke model, i.e. the multiqubit two-photon Rabi model, retains the special feature of spectral collapse, and that adding more qubits lowers the critical value of the individual qubit coupling by a factor of *N*, where *N* denotes the number of qubits present. The spectral collapse can thus be realized more easily with the state-of-the-art circuit quantum electrodynamics technology. They also propose that the two-photon Dicke model could be realistically implemented using the trapped-ion technologies^27,28^ and some atomic or solid-state systems, e.g. superconducting devices in which bosonic modes have been coupled to spin assembles^29,30^. Nevertheless, a full understanding of the spectral collapse occuring in the two-photon Dicke model is still lacking.

Accordingly, it is the aim of our work to scrutinize the spectral collapse of the two-photon Dicke model, particularly those special cases involving several qubits only. The critical value of the coupling strength of the two-photon Dicke model with two (three) qubits is found to be half (one-third) of that of the two-photon Rabi model. To determine the discrete eigenenergies at the critical coupling for both of the two-qubit and three-qubit cases, we simply map the problem into the bound state problem involving a particle of variable effective mass in the presence of a finite potential well and a nonlocal potential, whose discrete eigenspectrum can be easily obtained by an elementary quantum mechanics approach. Similarly, the problem of determining the continuous eigenenergy spectrum at the critical coupling can also be mapped into the scattering state problem associated with a particle of variable effective mass, subject to both a local potential barrier and a nonlocal potential.

## Two-photon Dicke model

The two-photon Dicke model is described by the Hamiltonian $\left( \hbar =1\right)$^4^:

1 $$\begin{aligned} H=\omega _{0}\sum _{j=1}^{N}S_{jz}+\omega a^{\dag }a+2\epsilon \left( a^{\dag 2}+a^{2}\right) \sum _{j=1}^{N}S_{jx}, \end{aligned}$$

where $\omega$ is the frequency of the radiation mode specified by the bosonic operators *a* and $a^{\dag }$, each pair of atomic levels (or qubit) separated by an energy difference $\omega _{0}$ are represented by the spin-half operators $S_{jz}$ and $S_{jx}$ for $j=1,2,3,\ldots ,N$, and the atom-field coupling strength of each qubit is measured by the positive parameter $\epsilon$. The total spin operator ${\vec{\mathbf{S}}}\equiv \sum _{j=1}^{N}{\vec{S}}_{j}$ can assume *N*/2 different spin values, namely $\left\{ 0,1,2,\ldots ,N/2\right\}$ for *N* being even and $\left\{ 1/2,3/2,5/2,\ldots ,N/2\right\}$ for *N* being odd. It should be noted that in the spin-zero case the eigenenergies of *H* are simply given by $E_{n}=\omega n$ for $n=0,1,2,\ldots$, irrespective of the coupling strength, and they are called the “trapping states” or “dark states”^31^. All the other spin sectors, however, exhibit the special feature of spectral collapse. The critical value of the single-qubit coupling strength can be determined as follows.

For simplicity, we set the energy unit such that $\omega =1$ in the following analysis. By a spin rotation about the *y*-axis, the Hamiltonian *H* in Eq. (1) can be transformed into

2 $$\begin{aligned} {\tilde{H}}&= \omega _{0}\sum _{j=1}^{N}S_{jx}+a^{\dag }a-2\epsilon \left( a^{\dag 2}+a^{2}\right) \sum _{j=1}^{N}S_{jz} \nonumber \\&= \omega _{0} {\mathbf{S}}_{\mathbf{x}}+H_{0}-2\epsilon \left( x^{2}-p^{2}\right) {\mathbf{S}}_{\mathbf{z}}-\frac{1}{2}, \end{aligned}$$

for

3 $$\begin{aligned} x=\frac{1}{\sqrt{2}}\left( a+a^{\dag }\right) \quad {\text {and}}\quad p=\frac{1 }{i\sqrt{2}}\left( a-a^{\dag }\right) \end{aligned}$$

being the “position” and “momentum” operators of the boson mode, respectively. Here $H_{0}$ is the Hamiltonian of a quantum simple harmonic oscillator of unit mass and unit angular frequency. In the special case of $\omega _{0}=0$ the Hamiltonian ${\tilde{H}}$ is reduced to

4 $$\begin{aligned} {\tilde{H}}=\frac{1}{2}\left( 1+4\epsilon {\mathbf{S}}_{\mathbf{z}}\right) p^{2}+\frac{ 1}{2}\left( 1-4\epsilon {\mathbf{S}}_{\mathbf{z}}\right) x^{2}-\frac{1}{2}, \end{aligned}$$

whose eigenstates are simply given by the product states $\left\{ |M_{z}\rangle |\phi \rangle \right\}$, with $|M_{z}\rangle$ being an eigenstate of the total spin operator ${\mathbf{S}}_{\mathbf{z}}$ and $|\phi \rangle$ an eigenstate of the one-body Hamiltonian *h*:

5 $$\begin{aligned} h=\frac{1}{2}\left( 1+4\epsilon M_{z}\right) p^{2}+\frac{1}{2}\left( 1-4\epsilon M_{z}\right) x^{2}-\frac{1}{2}. \end{aligned}$$

Apparently, in each subspace of $M_{z}\ne 0$ there exists a critical value of the single-qubit coupling strength, namely $\epsilon _{c}\equiv 1/\left| 4M_{z}\right|$, implying the occurrence of the spectral collapse as in the two-photon Rabi model. For $\omega _{0}\ne 0$ the above analysis still holds for both $\epsilon <\epsilon _{c}$ and $\epsilon >\epsilon _{c}$ because the first term in Eq. (2) is a bounded operator. The characteristic feature of the incomplete spectral collapse at the critical coupling $\epsilon _{c}$, however, remains unclear. Likewise, since the maximum value of $\left| M_{z}\right|$ is *N*/2, occurring in the total spin sector of *N*/2, the smallest possible single-qubit critical coupling strength is given by $\epsilon _{c}\equiv 1/\left( 2N\right)$. The 1/*N* dependence of the critical coupling thus ensures that the spectral collapse can be achieved with relatively small coupling strength by adding more qubits^1,4^.

At the critical coupling $\epsilon _{c}\equiv 1/\left( 2N\right)$ of the total spin sector of *N*/2, Eq. (2) is reduced to

6 $$\begin{aligned} {\tilde{H}}=\omega _{0} {\mathbf{S}}_{\mathbf{x}}+\frac{1}{2}\left( 1+\frac{2}{N} {\mathbf{S}} _{\mathbf{z}}\right) p^{2}+\frac{1}{2}\left( 1-\frac{2}{N} {\mathbf{S}}_{\mathbf{z} }\right) x^{2}-\frac{1}{2}, \end{aligned}$$

where the total spin operators ${\mathbf{S}}_{\mathbf{z}}$ and ${\mathbf{S}}_{\mathbf{x}}$ are given by

7 $$\begin{aligned} {\mathbf{S}}_{\mathbf{z}}&= \left( \begin{array}{lllllll} \frac{N}{2} &\quad 0 &\quad 0 &\quad \cdots &\quad 0 &\quad 0 &\quad 0 \\ 0 &\quad \frac{N}{2}-1 &\quad 0 &\quad \cdots &\quad 0 &\quad 0 &\quad 0 \\ 0 &\quad 0 &\quad \frac{N}{2}-2 &\quad \cdots &\quad 0 &\quad 0 &\quad 0 \\ \vdots &\quad \vdots &\quad \vdots &\quad \vdots & \quad \vdots &\quad \vdots & \quad \vdots \\ 0 &\quad 0 &\quad 0 &\quad \cdots &\quad -\frac{N}{2}+2 &\quad 0 &\quad 0 \\ 0 &\quad 0 &\quad 0 &\quad \cdots &\quad 0 &\quad -\frac{N}{2}+1 &\quad 0 \\ 0 &\quad 0 &\quad 0 &\quad \cdots &\quad 0 &\quad 0 &\quad -\frac{N}{2} \end{array} \right) \end{aligned}$$

8 $$\begin{aligned} {\mathbf{S}}_{\mathbf{x}}&= \frac{1}{2}\left( \begin{array}{lllllll} 0 &\quad b_{\frac{N}{2}} &\quad 0 &\quad \cdots &\quad 0 &\quad 0 &\quad 0 \\ b_{\frac{N}{2}} &\quad 0 &\quad b_{\frac{N}{2}-1} &\quad \cdots &\quad 0 &\quad 0 &\quad 0 \\ 0 &\quad b_{\frac{N}{2}-1} &\quad 0 &\quad \cdots &\quad 0 &\quad 0 &\quad 0 \\ \vdots &\quad \vdots &\quad \vdots &\quad \vdots &\quad \vdots &\quad \vdots &\quad \vdots \\ 0 &\quad 0 &\quad 0 &\quad \cdots &\quad 0 &\quad b_{-\frac{N}{2}+2} &\quad 0 \\ 0 &\quad 0 &\quad 0 &\quad \cdots &\quad b_{-\frac{N}{2}+2} &\quad 0 &\quad b_{-\frac{N}{2}+1} \\ 0 &\quad 0 &\quad 0 &\quad \cdots &\quad 0 &\quad b_{-\frac{N}{2}+1} &\quad 0 \end{array} \right) \end{aligned}$$

for $b_{j}=\sqrt{\left( \frac{N}{2}+j\right) \left( \frac{N+2}{2}-j\right) }$. In the coordinate space the eigenvalue equation of ${\tilde{H}}$ reads

9 $$\begin{aligned} \left\{ \begin{array}{l} \left( -\frac{d^{2}}{dx^{2}}-E-\frac{1}{2}\right) \psi _{\frac{N}{2}}\left( x\right) =-\frac{\omega _{0}}{2}b_{\frac{N}{2}}\psi _{\frac{N}{2}-1}\left( x\right) \\ \left( -\frac{N-1}{N}\frac{d^{2}}{dx^{2}}+\frac{1}{N}x^{2}-E-\frac{1}{2} \right) \psi _{\frac{N}{2}-1}\left( x\right) =-\frac{\omega _{0}}{2}\left[ b_{\frac{N}{2}}\psi _{\frac{N}{2}}\left( x\right) +b_{\frac{N}{2}-1}\psi _{ \frac{N}{2}-2}\left( x\right) \right] \\ \left( -\frac{N-2}{N}\frac{d^{2}}{dx^{2}}+\frac{2}{N}x^{2}-E-\frac{1}{2} \right) \psi _{\frac{N}{2}-2}\left( x\right) =-\frac{\omega _{0}}{2}\left[ b_{\frac{N}{2}-1}\psi _{\frac{N}{2}-1}\left( x\right) +b_{\frac{N}{2}-2}\psi _{\frac{N}{2}-3}\left( x\right) \right] \\\quad\,\,\quad\quad\quad\quad\quad\quad\quad\quad\quad\quad\quad\quad \vdots\quad\quad\quad\quad\quad\quad \quad \vdots\quad\quad\quad\quad\quad\quad\quad \vdots \\ \left( -\frac{2}{N}\frac{d^{2}}{dx^{2}}+\frac{N-2}{N}x^{2}-E-\frac{1}{2} \right) \psi _{-\frac{N}{2}+2}\left( x\right) =-\frac{\omega _{0}}{2}\left[ b_{-\frac{N}{2}+3}\psi _{-\frac{N}{2}+3}\left( x\right) +b_{-\frac{N}{2} +2}\psi _{-\frac{N}{2}+1}\left( x\right) \right] \\ \left( -\frac{1}{N}\frac{d^{2}}{dx^{2}}+\frac{N-1}{N}x^{2}-E-\frac{1}{2} \right) \psi _{-\frac{N}{2}+1}\left( x\right) =-\frac{\omega _{0}}{2}\left[ b_{-\frac{N}{2}+2}\psi _{-\frac{N}{2}+2}\left( x\right) +b_{-\frac{N}{2} +1}\psi _{-\frac{N}{2}}\left( x\right) \right] \\ \left( x^{2}-E-\frac{1}{2}\right) \psi _{-\frac{N}{2}}\left( x\right) =- \frac{\omega _{0}}{2}b_{-\frac{N}{2}+1}\psi _{-\frac{N}{2}+1}\left( x\right) \end{array} \right. , \end{aligned}$$

where *E* denotes the eigenenergy. Beyond question it is a formidable task to solve this set of coupled 2nd-order ordinary differential equations. In order to gain insights of the behaviour of the two-photon Dicke model at the critical coupling, we shall examine two special cases, namely the two-qubit case and the three-qubit case, in the following two sections.

## Two-qubit case at the critical coupling

To begin with, we shall concentrate on the simplest case of two qubits, which involves a set of three coupled equations only:

10 $$\begin{aligned} \left\{ \begin{array}{l} \left( -\frac{d^{2}}{dx^{2}}-E-\frac{1}{2}\right) \psi _{1}\left( x\right) =- \frac{\omega _{0}}{\sqrt{2}}\psi _{0}\left( x\right) \\ \left( -\frac{1}{2}\frac{d^{2}}{dx^{2}}+\frac{1}{2}x^{2}-E-\frac{1}{2} \right) \psi _{0}\left( x\right) =-\frac{\omega _{0}}{\sqrt{2}}\left[ \psi _{1}\left( x\right) +\psi _{-1}\left( x\right) \right] \\ \left( x^{2}-E-\frac{1}{2}\right) \psi _{-1}\left( x\right) =-\frac{\omega _{0}}{\sqrt{2}}\psi _{0}\left( x\right) \end{array} \right. . \end{aligned}$$

For $E+1/2<0$ we define $\kappa =\sqrt{\left| E+1/2\right| }$ and $x=\kappa q$. Then $\psi _{-1}\left( x\right)$ and $\psi _{1}\left( x\right)$ can be easily determined as

11 $$\begin{aligned} \psi _{-1}\left( q\right)&= -\frac{\omega _{0}}{\sqrt{2}\kappa ^{2}}\left( \frac{1}{1+q^{2}}\right) \psi _{0}\left( q\right) \end{aligned}$$

12 $$\begin{aligned} \psi _{1}\left( q\right)&= -\frac{\omega _{0}}{2\sqrt{2}}\int _{-\infty }^{\infty }d\xi \exp \left\{ -\kappa ^{2}\left| \xi \right| \right\} \psi _{0}\left( q+\xi \right) . \end{aligned}$$

As a result, we are left with an integro-differential equation involving $\psi _{0}\left( q\right)$:

13 $$\begin{aligned} -\kappa ^{4}\left( 1+\frac{1}{2}q^{2}\right) \psi _{0}\left( q\right)&= - \frac{1}{2}\frac{d^{2}\psi _{0}\left( q\right) }{dq^{2}}-\frac{\omega _{0}^{2}}{2}\left( \frac{1}{1+q^{2}}\right) \psi _{0}\left( q\right) \nonumber \\&\quad -\frac{\kappa ^{2}\omega _{0}^{2}}{4}\int _{-\infty }^{\infty }d\xi \exp \left\{ -\kappa ^{2}\left| \xi \right| \right\} \psi _{0}\left( q+\xi \right) . \end{aligned}$$

By assuming that $\psi _{0}\left( q\right)$ takes the form

14 $$\begin{aligned} \psi _{0}\left( q\right) =\frac{1}{\sqrt{1+\frac{1}{2}q^{2}}}\phi _{0}\left( q\right) , \end{aligned}$$

it is straightforward to show that $\phi _{0}\left( q\right)$ satisfies

15 $$\begin{aligned} -\kappa ^{4}\phi _{0}\left( q\right)&= -\frac{1}{2}\left\{ \frac{1}{\sqrt{ M\left( q\right) }}\frac{d^{2}}{dq^{2}}\frac{1}{\sqrt{M\left( q\right) }} \right\} \phi _{0}\left( q\right) +V\left( q\right) \phi _{0}\left( q\right) \nonumber \\&\quad -\frac{\kappa ^{2}\omega _{0}^{2}}{4\sqrt{1+\frac{1}{2}q^{2}}}\int _{-\infty }^{\infty }d\xi U\left( q,\xi ;\kappa \right) \phi _{0}\left( q+\xi \right) , \end{aligned}$$

where

16 $$\begin{aligned} M\left( q\right)&= 1+\frac{q^{2}}{2} \end{aligned}$$

17 $$\begin{aligned} V\left( q\right)&= -\frac{\omega _{0}^{2}}{\left( 2+q^{2}\right) \left( 1+q^{2}\right) } \end{aligned}$$

18 $$\begin{aligned} U\left( q,\xi ;\kappa \right)&= \frac{\exp \left\{ -\kappa ^{2}\left| \xi \right| \right\} }{\sqrt{1+\frac{1}{2}\left( q+\xi \right) ^{2}}}. \end{aligned}$$

This is the time-independent Schrödinger equation of the bound state problem associated with a particle of variable effective mass $M\left( q\right)$ in the presence of a finite potential well $V\left( q\right)$ and a nonlocal potential $U\left( q,\xi ;\kappa \right)$^32,33^.

For $\kappa \ll 1$, namely the cases of shallow bound states, we can approximate the integral on the right-hand side of Eq. (15) by

19 $$\begin{aligned} \int _{-\infty }^{\infty }d\xi U\left( q,\xi ;\kappa \right) \phi _{0}\left( q+\xi \right) \approx \int \limits _{-\infty }^{\infty }d\xi \psi _{0}\left( \xi \right) . \end{aligned}$$

Provided that $\psi _{0}\left( \xi \right)$ is normalizable, the integral gives a constant *C*. The term involving the nonlocal potential becomes independent of $\phi _{0}\left( q\right)$:

$$\begin{aligned} -\frac{\kappa ^{2}\omega _{0}^{2}}{4\sqrt{1+\frac{1}{2}q^{2}}}. \end{aligned}$$

As $\kappa \ll 1$, the term involving the nonlocal potential can be neglected, and thus Eq. (15) is reduced to

20 $$\begin{aligned} -\kappa ^{4}\phi _{0}\left( q\right) =-\frac{1}{2}\left\{ \frac{1}{\sqrt{ M\left( q\right) }}\frac{d^{2}}{dq^{2}}\frac{1}{\sqrt{M\left( q\right) }} \right\} \phi _{0}\left( q\right) +V\left( q\right) \phi _{0}\left( q\right) , \end{aligned}$$

which is the time-independent Schrödinger equation of the bound state problem associated with a particle of variable effective mass $M\left( q\right)$ in the finite potential well $V\left( q\right)$. Hence, we have demonstrated the existence of shallow bound states of the two-photon Dicke model with two qubits.

Moreover, the existence of these shallow bound states implies that lower bound states may also exist. For $\omega _{0}\gg 1$ and $\kappa \lesssim \omega _{0}$, namely the cases of low-lying bound states, we approximate the integral on the right-hand side of Eq. (15) by

21 $$\begin{aligned} \int _{-\infty }^{\infty }d\xi U\left( q,\xi ;\kappa \right) \phi _{0}\left( q+\xi \right)&= \psi _{0}\left( q\right) \int \limits _{-\infty }^{\infty }d\xi \exp \left\{ -\kappa ^{2}\left| \xi \right| \right\} \nonumber \\&\quad +\sum _{n=1}^{\infty }\frac{d^{2n}\psi _{0}\left( q\right) }{dq^{2n}} \int \limits _{-\infty }^{\infty }d\xi \exp \left\{ -\kappa ^{2}\left| \xi \right| \right\} \xi ^{2n} \nonumber \\&\approx \frac{2}{\kappa ^{2}}\psi _{0}\left( q\right) +\frac{2}{\kappa ^{6} }\frac{d^{2}\psi _{0}\left( q\right) }{dq^{2}}+\frac{2}{\kappa ^{10}}\frac{ d^{4}\psi _{0}\left( q\right) }{dq^{4}}+\cdots \cdots \end{aligned}$$

If only the leading-order term is kept, then Eq. (15) can be simplified to yield

22 $$\begin{aligned} -\kappa ^{4}\phi _{0}\left( q\right) =-\frac{1}{2}\left\{ \frac{1}{\sqrt{ M\left( q\right) }}\frac{d^{2}}{dq^{2}}\frac{1}{\sqrt{M\left( q\right) }} \right\} \phi _{0}\left( q\right) -\frac{\omega _{0}^{2}}{1+q^{2}}\phi _{0}\left( q\right) , \end{aligned}$$

which is the time-independent Schrödinger equation of the bound state problem associated with a particle of variable effective mass $M\left( q\right)$ trapped in a “Lorentzian function” potential well. Accordingly, the existence of low-lying bound states has been confirmed.

On the other hand, for $E+1/2>0$ we define $k=\sqrt{E+1/2}$ and $x=k{\bar{q}}$. Then the three coupled equations in Eq. (10) can be rewritten as

23 $$\begin{aligned} \psi _{-1}\left( {\bar{q}}\right)&= \frac{\omega _{0}}{\sqrt{2}k^{2}}\left( \frac{1}{1-{\bar{q}}^{2}}\right) \psi _{0}\left( {\bar{q}}\right) \end{aligned}$$

24 $$\begin{aligned} \psi _{1}\left( {\bar{q}}\right)&= \frac{\omega _{0}}{\sqrt{2}}\int _{-\infty }^{\infty }\frac{d{\bar{\xi }}}{2\pi }\psi _{0}\left( {\bar{q}}+{\bar{\xi }}\right) \int _{-\infty }^{\infty }d\mu \frac{\exp \left\{ ik^{2}\mu {\bar{\xi }}\right\} }{1-\mu ^{2}+i\eta } \end{aligned}$$

25 $$\begin{aligned} k^{4}\left( 1-\frac{1}{2}{\bar{q}}^{2}\right) \psi _{0}\left( {\bar{q}}\right)&= -\frac{1}{2}\frac{d^{2}\psi _{0}\left( {\bar{q}}\right) }{d{\bar{q}}^{2}}+ \frac{\omega _{0}^{2}}{2}\left( \frac{1}{1-{\bar{q}}^{2}}\right) \psi _{0}\left( {\bar{q}}\right) \nonumber \\&\quad +\frac{k^{2}\omega _{0}^{2}}{4\pi }\int _{-\infty }^{\infty }d{\bar{\xi }}\psi _{0}\left( {\bar{q}}+{\bar{\xi }}\right) \int _{-\infty }^{\infty }d\mu \frac{\exp \left\{ ik^{2}\mu {\bar{\xi }}\right\} }{1-\mu ^{2}+i\eta }, \end{aligned}$$

where it is understood that the limiting process $\eta \rightarrow 0^{+}$ is being taken after the evaluation of the integral over $\mu$. Again, if $\psi _{0}\left( {\bar{q}}\right)$ assumes the form

26 $$\begin{aligned} \psi _{0}\left( {\bar{q}}\right) =\frac{1}{\sqrt{1-\frac{1}{2}{\bar{q}}^{2}}} \phi _{0}\left( {\bar{q}}\right) , \end{aligned}$$

then $$\phi _{0}\left( {\bar{q}}\right)$$ obeys

27 $$\begin{aligned} k^{4}\phi _{0}\left( {\bar{q}}\right)&= -\frac{1}{2}\left\{ \frac{1}{\sqrt{ {\bar{M}}\left( {\bar{q}}\right) }}\frac{d^{2}}{d{\bar{q}}^{2}}\frac{1}{\sqrt{\bar{ M}\left( {\bar{q}}\right) }}\right\} \phi _{0}\left( {\bar{q}}\right) +{\bar{V}} \left( {\bar{q}}\right) \phi _{0}\left( {\bar{q}}\right) \nonumber \\&\quad +\frac{k^{2}\omega _{0}^{2}}{4\pi }\frac{1}{\sqrt{1-\frac{1}{2}{\bar{q}}^{2}}} \int _{-\infty }^{\infty }d{\bar{\xi }}{\bar{U}}\left( {\bar{q}},{\bar{\xi }};\kappa \right) \phi _{0}\left( {\bar{q}}+{\bar{\xi }}\right) \end{aligned}$$

where

28 $$\begin{aligned} {\bar{M}}\left( {\bar{q}}\right)&= 1-\frac{{\bar{q}}^{2}}{2} \end{aligned}$$

29 $$\begin{aligned} {\bar{V}}\left( {\bar{q}}\right)&= \frac{\omega _{0}^{2}}{\left( 2-{\bar{q}} ^{2}\right) \left( 1-{\bar{q}}^{2}\right) } \end{aligned}$$

30 $$\begin{aligned} {\bar{U}}\left( {\bar{q}},{\bar{\xi }};\kappa \right)&= \frac{1}{\sqrt{1-\frac{1}{2 }\left( {\bar{q}}+{\bar{\xi }}\right) ^{2}}}\int _{-\infty }^{\infty }d\mu \frac{ \exp \left\{ ik^{2}\mu {\bar{\xi }}\right\} }{1-\mu ^{2}+i\eta }. \end{aligned}$$

It is not difficult to recognize that Eq. (27) is the time-independent Schrödinger equation of the scattering state problem associated with a particle of variable effective mass, subject to both a local potential barrier and a nonlocal potential. Accordingly, for $E+1/2>0$ the eigenstates are not normalizable and a continuous eigenenergy spectrum is expected.

## Three-qubit case at the critical coupling

Similarly, in the case of three qubits we need to solve a set of four coupled equations:

31 $$\begin{aligned} \left\{ \begin{array}{l} \left( -\frac{d^{2}}{dx^{2}}-E-\frac{1}{2}\right) \psi _{\frac{3}{2}}\left( x\right) =-\frac{\sqrt{3}\omega _{0}}{2}\psi _{\frac{1}{2}}\left( x\right) \\ \left( -\frac{2}{3}\frac{d^{2}}{dx^{2}}+\frac{1}{3}x^{2}-E-\frac{1}{2} \right) \psi _{\frac{1}{2}}\left( x\right) =-\omega _{0}\left[ \frac{\sqrt{3} }{2}\psi _{\frac{3}{2}}\left( x\right) +\psi _{-\frac{1}{2}}\left( x\right) \right] \\ \left( -\frac{1}{3}\frac{d^{2}}{dx^{2}}+\frac{2}{3}x^{2}-E-\frac{1}{2} \right) \psi _{-\frac{1}{2}}\left( x\right) =-\omega _{0}\left[ \frac{\sqrt{3 }}{2}\psi _{-\frac{3}{2}}\left( x\right) +\psi _{\frac{1}{2}}\left( x\right) \right] \\ \left( x^{2}-E-\frac{1}{2}\right) \psi _{-\frac{3}{2}}\left( x\right) =- \frac{\sqrt{3}\omega _{0}}{2}\psi _{-\frac{1}{2}}\left( x\right) \end{array} \right. , \end{aligned}$$

Performing the Fourier transform to the first two equations yields

32 $$\begin{aligned} \left\{ \begin{array}{l} \left( p^{2}-E-\frac{1}{2}\right) {\tilde{\psi }}_{\frac{3}{2}}\left( p\right) =-\frac{\sqrt{3}\omega _{0}}{2}{\tilde{\psi }}_{\frac{1}{2}}\left( p\right) \\ \left( -\frac{1}{3}\frac{d^{2}}{dp^{2}}+\frac{2}{3}p^{2}-E-\frac{1}{2} \right) {\tilde{\psi }}_{\frac{1}{2}}\left( p\right) =-\omega _{0}\left[ \frac{ \sqrt{3}}{2}{\tilde{\psi }}_{\frac{3}{2}}\left( p\right) +{\tilde{\psi }}_{-\frac{1 }{2}}\left( p\right) \right] \end{array} \right. , \end{aligned}$$

where ${\tilde{\psi }}_{\nu }\left( p\right)$ is the Fourier transform of $\psi _{\nu }\left( x\right)$:

33 $$\begin{aligned} {\tilde{\psi }}_{\nu }\left( p\right) =\int _{-\infty }^{\infty }\frac{dx}{\sqrt{ 2\pi }}e^{ipx}\psi _{\nu }\left( x\right) . \end{aligned}$$

It is obvious that ${\tilde{\psi }}_{\frac{3}{2}}\left( p\right)$ and $\psi _{- \frac{3}{2}}\left( x\right)$ can be readily determined as

34 $$\begin{aligned} {\tilde{\psi }}_{\frac{3}{2}}\left( p\right)&= \frac{\frac{\sqrt{3}}{2}\omega _{0}}{E+\frac{1}{2}-p^{2}}{\tilde{\psi }}_{\frac{1}{2}}\left( p\right) \end{aligned}$$

35 $$\begin{aligned} \psi _{-\frac{3}{2}}\left( x\right)&= \frac{\frac{\sqrt{3}}{2}\omega _{0}}{ E+\frac{1}{2}-x^{2}}\psi _{-\frac{1}{2}}\left( x\right) . \end{aligned}$$

Thus, we are left with two coupled equations connecting $\psi _{\frac{1}{2} }\left( x\right)$ and $\psi _{-\frac{1}{2}}\left( x\right)$ as follows:

36 $$\begin{aligned} \left\{ \begin{array}{l} \left[ -\frac{1}{3}\frac{d^{2}}{dp^{2}}+\frac{2}{3}p^{2}+\frac{\left( 3/4\right) \omega _{0}^{2}}{E+\left( 1/2\right) -p^{2}}-\left( E+\frac{1}{2} \right) \right] {\tilde{\psi }}_{\frac{1}{2}}\left( p\right) =-\omega _{0} {\tilde{\psi }}_{-\frac{1}{2}}\left( p\right) \\ \left[ -\frac{1}{3}\frac{d^{2}}{dx^{2}}+\frac{2}{3}x^{2}+\frac{\left( 3/4\right) \omega _{0}^{2}}{E+\left( 1/2\right) -x^{2}}-\left( E+\frac{1}{2} \right) \right] \psi _{-\frac{1}{2}}\left( x\right) =-\omega _{0}\psi _{ \frac{1}{2}}\left( x\right) \end{array} \right. . \end{aligned}$$

In terms of the sum and difference of the two wave functions ${\tilde{\psi }}_{ \frac{1}{2}}\left( x\right)$ and $\psi _{-\frac{1}{2}}\left( x\right)$:

$$\begin{aligned} \phi _{\pm }\left( x\right) ={\tilde{\psi }}_{\frac{1}{2}}\left( x\right) \pm \psi _{-\frac{1}{2}}\left( x\right) , \end{aligned}$$

the two coupled equations can be reduced to

37 $$\begin{aligned} \left\{ -\frac{1}{3}\frac{d^{2}}{dx^{2}}+\frac{2}{3}x^{2}+\frac{3\omega _{0}^{2}}{4\left( E+\frac{1}{2}-x^{2}\right) }-\left( E+\frac{1}{2}\right) \right\} \phi _{\pm }\left( x\right) =\pm \omega _{0}{\tilde{\phi }}_{\pm }\left( x\right) , \end{aligned}$$

where ${\tilde{\phi }}_{\pm }\left( p\right)$ is the Fourier transform of $\phi _{\pm }\left( x\right)$:

38 $$\begin{aligned} {\tilde{\phi }}_{\pm }\left( p\right) =\int _{-\infty }^{\infty }\frac{dx}{\sqrt{ 2\pi }}e^{ipx}\phi _{\pm }\left( x\right) . \end{aligned}$$

For $E+1/2<0$ we define $\kappa =\sqrt{\left| E+1/2\right| }$ and $x=\kappa q$. Then Eq. (37) can be rewritten as

39 $$\begin{aligned} -\kappa ^{4}\phi _{\pm }\left( q\right)&= \frac{1}{\frac{3}{2}+q^{2}}\left[ -\frac{1}{2}\frac{d^{2}}{dq^{2}}-\frac{9\omega _{0}^{2}}{8\left( 1+q^{2}\right) }\right] \phi _{\pm }\left( q\right) \nonumber \\&\quad \pm \frac{1}{2}\sqrt{\frac{3}{2}}\omega _{0}\kappa ^{5}\int _{-\infty }^{\infty }\frac{d\xi }{\sqrt{2\pi }}e^{iq\xi \kappa ^{2}}\int _{-\infty }^{\infty }d\chi \phi _{\pm }\left( \xi +\chi \right) \exp \left\{ -\sqrt{\frac{3}{2}} \kappa ^{2}\left| \chi \right| \right\} . \end{aligned}$$

By assuming that $\phi _{\pm }\left( q\right)$ takes the form

40 $$\begin{aligned} \phi _{\pm }\left( q\right) =\frac{1}{\sqrt{\frac{3}{2}+q^{2}}}\varphi _{\pm }\left( q\right) , \end{aligned}$$

it is straightforward to show that $\varphi _{\pm }\left( q\right)$ satisfies

41 $$\begin{aligned} -\kappa ^{4}\varphi _{\pm }\left( q\right)&= -\frac{1}{2}\left\{ \frac{1}{ \sqrt{M\left( q\right) }}\frac{d^{2}}{dq^{2}}\frac{1}{\sqrt{M\left( q\right) }}\right\} \varphi _{\pm }\left( q\right) +V\left( q\right) \varphi _{\pm }\left( q\right) \nonumber \\&\quad \pm \frac{1}{2}\sqrt{\frac{3}{2}}\frac{\omega _{0}\kappa ^{5}}{\sqrt{\frac{3}{2 }+q^{2}}}\int _{-\infty }^{\infty }\frac{d\xi }{\sqrt{2\pi }}e^{iq\xi \kappa ^{2}}\int _{-\infty }^{\infty }d\chi U\left( \xi ,\chi ;\kappa \right) \varphi _{\pm }\left( \xi +\chi \right) , \end{aligned}$$

where

42 $$\begin{aligned} M\left( q\right)&= \frac{3}{2}+q^{2} \end{aligned}$$

43 $$\begin{aligned} V\left( q\right)&= -\frac{9\omega _{0}^{2}}{8\left( \frac{3}{2} +q^{2}\right) \left( 1+q^{2}\right) } \end{aligned}$$

44 $$\begin{aligned} U\left( \xi ,\chi ;\kappa \right)&= \frac{\exp \left\{ -\sqrt{\frac{3}{2}} \kappa ^{2}\left| \chi \right| \right\} }{\sqrt{\frac{3}{2}+\left( \xi +\chi \right) ^{2}}}. \end{aligned}$$

Accordingly, we have derived the time-independent Schrödinger equation of the bound state problem associated with a particle of variable effective mass $M\left( q\right)$ subject to a finite potential well $V\left( q\right)$ and a nonlocal potential $U\left( \xi ,\chi ;\kappa \right)$^32,33^.

For $\kappa \ll 1$, namely the cases of shallow bound states, the integral over the variable $\chi$ on the right-hand side of Eq. (41) can be approximated by

45 $$\begin{aligned} \int _{-\infty }^{\infty }d\chi U\left( \xi ,\chi ;\kappa \right) \varphi _{\pm }\left( \xi +\chi \right) \approx \int \limits _{-\infty }^{\infty }d\chi \phi _{\pm }\left( \chi \right) . \end{aligned}$$

The integral gives a constant *C* provided that $\phi _{\pm }\left( \chi \right)$ is normalizable. The term involving the nonlocal potential can then be analytically evaluated to give

$$\begin{aligned} \pm \sqrt{\frac{\pi }{2}}C\omega _{0}\kappa ^{3}\delta \left( q\right) , \end{aligned}$$

which can be neglected for $\kappa \ll 1$. Hence, Eq. (41) is reduced to

46 $$\begin{aligned} -\kappa ^{4}\varphi _{\pm }\left( q\right) =-\frac{1}{2}\left\{ \frac{1}{ \sqrt{M\left( q\right) }}\frac{d^{2}}{dq^{2}}\frac{1}{\sqrt{M\left( q\right) }}\right\} \varphi _{\pm }\left( q\right) +V\left( q\right) \varphi _{\pm }\left( q\right) , \end{aligned}$$

which is the time-independent Schrödinger equation of the bound state problem associated with a particle of variable effective mass $M\left( q\right)$ in the finite potential well $V\left( q\right)$. As a result, we have demonstrated that the two-photon Dicke model with three qubits has a discrete energy spectrum for the existence of these shallow bound states implies that lower bound states may also exist.

On the other hand, for $E+1/2>0$ we define $k=\sqrt{E+1/2}$ and $x=k{\bar{q}}$. Then Eq. (37) becomes

47 $$\begin{aligned} k^{4}\phi _{\pm }\left( {\bar{q}}\right)&= \frac{1}{\frac{3}{2}-{\bar{q}}^{2}} \left[ -\frac{1}{2}\frac{d^{2}}{d{\bar{q}}^{2}}+\frac{9\omega _{0}^{2}}{ 8\left( 1-{\bar{q}}^{2}\right) }\right] \phi _{\pm }\left( {\bar{q}}\right) \nonumber \\&\quad \pm \frac{3}{4\pi }\omega _{0}k^{5}\int _{-\infty }^{\infty }\frac{d{\bar{\xi }}}{ \sqrt{2\pi }}e^{i{\bar{q}}{\bar{\xi }}k^{2}}\int _{-\infty }^{\infty }d{\bar{\chi }} \phi _{\pm }\left( {\bar{\xi }}+{\bar{\chi }}\right) \ \int _{-\infty }^{\infty }d\gamma \frac{e^{i{\bar{\chi }}\gamma k^{2}}}{\frac{3}{2}-\gamma ^{2}+i\eta }, \end{aligned}$$

where the limiting process $\eta \rightarrow 0^{+}$ is being taken after the evaluation of the integral over $\gamma$. By assuming that

48 $$\begin{aligned} \phi _{\pm }\left( {\bar{q}}\right) =\frac{1}{\sqrt{\frac{3}{2}-{\bar{q}}^{2}}} \varphi _{\pm }\left( {\bar{q}}\right) , \end{aligned}$$

Eq. (47) is reduced to

49 $$\begin{aligned} k^{4}\varphi _{\pm }\left( {\bar{q}}\right)&= -\frac{1}{2}\left\{ \frac{1}{ \sqrt{{\bar{M}}\left( {\bar{q}}\right) }}\frac{d^{2}}{d{\bar{q}}^{2}}\frac{1}{ \sqrt{{\bar{M}}\left( {\bar{q}}\right) }}\right\} \varphi _{\pm }\left( {\bar{q}} \right) +{\bar{V}}\left( {\bar{q}}\right) \varphi _{\pm }\left( {\bar{q}}\right) \nonumber \\&\quad \pm \frac{3\omega _{0}k^{5}}{4\pi }\frac{1}{\sqrt{\frac{3}{2}-{\bar{q}}^{2}}} \int _{-\infty }^{\infty }\frac{d{\bar{\xi }}}{\sqrt{2\pi }}e^{i{\bar{q}}{\bar{\xi }} k^{2}}\int _{-\infty }^{\infty }d{\bar{\chi }}\varphi _{\pm }\left( {\bar{\xi }}+ {\bar{\chi }}\right) \ {\bar{U}}\left( {\bar{\xi }},{\bar{\chi }};k\right) , \end{aligned}$$

where

50 $$\begin{aligned} {\bar{M}}\left( {\bar{q}}\right)&= \frac{3}{2}-{\bar{q}}^{2} \end{aligned}$$

51 $$\begin{aligned} {\bar{V}}\left( {\bar{q}}\right)&= \frac{9\omega _{0}^{2}}{8\left( \frac{3}{2}- {\bar{q}}^{2}\right) \left( 1-{\bar{q}}^{2}\right) } \end{aligned}$$

52 $$\begin{aligned} {\bar{U}}\left( {\bar{\xi }},{\bar{\chi }};k\right)&= \frac{1}{\sqrt{\frac{3}{2} -\left( {\bar{\xi }}+{\bar{\chi }}\right) ^{2}}}\int _{-\infty }^{\infty }d\gamma \frac{e^{i{\bar{\chi }}\gamma k^{2}}}{\frac{3}{2}-\gamma ^{2}+i\eta }. \end{aligned}$$

Hence, we have derived the time-independent Schrödinger equation of the scattering state problem associated with a particle of variable effective mass, subject to both a local potential barrier and a nonlocal potential, implying that for $E+1/2>0$ the eigenstates are not normalizable and a continuous eigenenergy spectrum is expected.

## Conclusion

In this work we have shown that the smallest possible single-qubit critical coupling strength $\epsilon _{c}$ of the N-qubit two-photon Rabi model is only 1/*N* times that of the two-photon Rabi model. The spectral collapse can thus occur at a more attainable value of the critical coupling^1,4^. For both of the two-qubit and three-qubit cases, we have also rigorously demonstrated that at the critical coupling $\epsilon _{c}$ the system exhibits an incomplete spectral collapse, namely having both a set of discrete eigenenergies and a continuous energy spectrum, and that the energy difference $\omega _{0}$ of each qubit determines the number of bound states available, implying that the extent of the incomplete spectral collapse can be monitored in a straightforward manner. Interestingly, the set of discrete eigenenergies can be derived from the bound state problem of a particle of variable effective mass in the presence of a finite potential well and a nonlocal potential, whilst the problem of determining the continuous eigenenergy spectrum can be mapped into the scattering state problem associated with a particle of variable effective mass, subject to both a local potential barrier and a nonlocal potential. Furthermore, as shown in Lo^25^, we may introduce a suitable unitary transformation to cast Eqs. (20), (22) and (46) into the time-independent Schrödinger equation of the bound state problem with a particle of unit mass moving in a finite potential well.

Finally, we would like to thank an anonymous referee for bringing a recent paper on the bound states of the two-photon Rabi model at the critical coupling to our attention^34^. The paper investigates the discrete eigenenergy levels via solving a time-independent Schrödinger equation with an energy-dependent effective potential, whose solutions are found by self-consistent iterative numerical calculations. This is in sharp constrast to the findings reported in Ref.^24^, which explicitly shows that the discrete eigenenergy spectrum has a one-to-one mapping with that of a particle in a “Lorentzian function” potential well, and that each eigenenergy is doubly degenerate. Particularly, Ref.^34^ shows no sign of the two-fold degeneracy. Perhaps it may be due to some flaws in the compicated numerical calculations or the entire formulation. All in all, similar to Ref.^24^, the present work is capable of providing a clear and concise treatment of the incomplete spectral collapse of the multi-qubit two-photon Rabi model at the critical coupling.
